# Applications and limitations of AI tools in enzyme design

**DOI:** 10.1002/pro.70698

**Published:** 2026-07-10

**Authors:** Rosa Teijeiro‐Juiz, Nina Egeler, Grzegorz Jamróg, Marion Ringel, Thomas Brück, Bruno Di Geronimo, Bernhard Loll

**Affiliations:** ^1^ Institute of Chemistry and Biochemistry, Laboratory of Structural Biochemistry Freie Universität Berlin Berlin Germany; ^2^ Werner Siemens‐Chair of Synthetic Biotechnology Technical University of Munich (TUM), TUM School of Natural Sciences Garching Germany; ^3^ School of Chemistry and Biochemistry Georgia Institute of Technology Atlanta Georgia USA

**Keywords:** artificial intelligence, de novo design, enzyme design, non‐canonical amino acids, protein engineering, protein stability

## Abstract

Enzymes catalyze various different chemical reactions often with high efficiency and selectivity compared to synthetic catalysts. Advances in protein engineering over the past decades have allowed researchers to design enzymes, improving their catalytic performance and adapting them for specific or entirely novel chemical reactions. However, the need for the experimental validation of thousands of computational designs remains one of the major bottlenecks. Yet another restriction is our still limited knowledge about transition state architectures, effects of mutations, active‐site dynamics just to name a few. To overcome this, the combination of experimental and computational methods is essential, yet many experimentalists are facing significant obstacles when entering the field of computational enzyme design. To address these obstacles, this review offers a comprehensive introduction and overview of several current artificial intelligence (AI)‐driven methods available for enzyme design, with a focus on reaction‐to‐sequence design, structure prediction, substrate scope prediction, engineering of stable variants, design of enzymes with non‐canonical amino acids, and de novo design. Subsequently, this work serves as an accessible guide for experimental researchers with interest in learning how to use AI‐based computational methods in enzyme engineering.

## 1 INTRODUCTION

Throughout evolution, nature has optimized metabolite and natural product transformations, giving rise to enzymes, highly efficient and selective catalytic proteins (Alves et al., 2002; Hossack et al., 2023; Mori et al., 2025; Ribeiro et al., 2023). The applications of enzymes encompass but are not limited to food, biopharmaceutical, and chemical applications (Karigar & Rao, 2011; Meghwanshi et al., 2020; Robinson, 2015; Welborn & Head‐Gordon, 2019).

A major current focus in the field of protein science is target‐oriented enzyme engineering, which aims to tailor enzymes' activities to perform substrate‐specific or non‐natural chemical reactions (Hossack et al., 2023). Historically, a deep structural understanding of enzymes was required to select specific modifications in the active site which would allow for the optimized catalysis of natural substrates, or the accommodation of alternative, non‐natural substrate analogs (Yang et al., 2024). In contrast to directed evolution approaches, which often require experimentally testing thousands of variants, structure‐based design targets a smaller library of enzyme variants, cutting down the time needed for optimization (Verma et al., 2015; Wijma & Janssen, 2013). However, the need for quality structural data has traditionally limited this approach (Chen et al., 2025).

More recently, computational techniques such as molecular docking, molecular dynamics (MD) simulations, and quantum mechanics (QM) have emerged as an additional step in modern enzyme engineering campaigns (Ferreira et al., 2022; Sun et al., 2024). The integration of machine learning (ML) and deep learning now enables accurate prediction of structure–function relationships and the design of new catalytic scaffolds with remarkable precision and speed (Wang et al., 2025). However, these methods are computationally expensive and require a deep understanding of computational biology.

Newly developed artificial intelligence (AI)‐based tools have revolutionized our understanding of protein structure and function, opening new possibilities for rational, data‐driven enzyme design. These automated methods are based on large databases, allowing non‐experts to also make use of them and easily tailor enzyme properties such as thermal stability, substrate specificity, or activity (Hossack et al., 2023; Singh et al., 2025). During the last years, AI‐based tools have helped come up with a completely new way of understanding enzyme engineering: de novo design, which seeks to create new enzymes, that will be able to catalyze a reaction of interest (Cui, Su, et al., 2025; Cui, Zheng, et al., 2025a, 2025b).

More general reviews introducing the methodology of protein folding and design have been recently published (Albanese et al., 2025; Ferruz & Höcker, 2022; Koh et al., 2025; Kosonocky et al., 2026; Listov et al., 2025; Stocco et al., 2026). Multiple reviews describing AI‐enzyme design methods have been published, providing comprehensive overviews of the available tools and their applications. In this review, we instead aim to provide a practical and critical guide for experimental researchers interested in applying computational techniques but uncertain about where to begin.

Hereby, in this work we focus on a curated subset of methods across key areas for enzyme design campaigns, including reaction‐to‐sequence design, structure prediction, substrate specificity, protein stability, use of non‐canonical amino acids (ncAAs), and de novo design. Given the broad scope of the field, this review does not aim to be exhaustive; rather, we highlight representative approaches with demonstrated relevance to computational‐experimental workflows and discuss their strengths and limitations, including their experimental validation.

Readers seeking a more comprehensive description of methods should refer to previous reviews, such as those by Wen et al. (2025), which focuses on generative models, and Wang et al. (2025), which also covers tools for enzyme function and enzyme kinetics prediction. For methodologies which constitute main steps in enzyme design campaigns but are not AI‐based, readers should refer to the cited literature. These include established computational tools for protein design and analysis such as PROSS (Weinstein et al., 2021), FuncLib (Khersonsky et al., 2018), FoldX (Schymkowitz et al., 2005), and MD workflows (Hollingsworth & Dror, 2018; Tuckerman & Martyna, 2000).

## 2 AI FOR REACTION‐TO‐SEQUENCE DESIGN

Designing enzymes based on a desired chemical reaction involves mapping from chemistry to protein sequences. This can be approached through two complementary AI paradigms: (i) predictive models, which evaluate or rank existing natural enzyme sequences for a target reaction, and (ii) generative models, which create novel sequences conditioned on catalytic function or reaction descriptors. This distinction is important because many currently available tools are highly effective for candidate prioritization but do not directly generate new sequences. In practice, predictive models often represent the first step of enzyme design workflows, where natural homologs or sequence libraries are screened before experimental testing, while generative approaches aim to expand sequence space beyond naturally occurring enzymes. Deep‐learning methods, including transformers and Large Language Models (LLMs), are increasingly being applied to both tasks by learning relationships between reactions, substrates, structures, and protein sequences from public datasets (Cui et al., 2025a; Cui, Zheng, et al., 2025a, 2025b). In this section, we summarize representative tools in both categories (Table 1).

**TABLE 1:** Artificial intelligence‐driven methods for reaction‐to‐sequence design. More details about each model are provided in Table S1.

Model	Description	Experimental validation	Repository/webserver	References
SelenzymeRF	Evaluation of enzymes for biochemical reactions and metabolic pathway design	Yes	http://selenzymeRF.synbiochem.co.uk/ https://github.com/synbiochem/selenzyme	Stoney et al. (2023)
ESP	Prediction of interactions between enzyme sequences and substrates	No	https://esp.cs.hhu.de/ https://github.com/AlexanderKroll/ESP	Kroll et al. (2023)
ProSmith	Prediction of enzyme–substrate interactions	No	https://github.com/AlexanderKroll/ProSmith	Kroll et al. (2023)
FusionESP	Investigates substrate scope of an enzyme	No	https://github.com/dzjxzyd/FusionESP	Du et al. (2025)
ALDELE	Predicts if an enzyme will catalyze a reaction	No	https://github.com/Xiangwen‐Wang/ALDELE	Wang et al. (2024)
CATNIP	Matches substrates to enzymes in α‐ketoglutarate/Fe(II) non‐heme iron family	No	https://catnip.cheme.cmu.edu/	Paton et al. (2025)
CLEAN	Enzyme function prediction	Yes	https://github.com/tttianhao/CLEAN	Yu et al. (2023)
EZSpecifity	Predicts substrates an enzyme is likely to act on	No	https://zenodo.org/records/17981381	Cui, Su, et al. (2025), Cui, Zheng, et al. (2025a, 2025b)

Abbreviations: CLEAN, Contrastive learning‐enabled enzyme annotation; ESP, Enzyme–Substrate Predictor.

The detailed features, coverage, and curation of databases supporting these AI tools are beyond the scope of this review and have been summarized elsewhere (Presern & Golicnik, 2023). Here, we list representative resources: BRENDA (Chang et al., 2021), UniProt (UniProt, 2025), Kyoto encyclopedia of genes and genomes (Kanehisa & Goto, 2000)), MetaCyc (Caspi et al., 2020), Expasy ENZYME (Bairoch, 2000), system for the analysis of biochemical pathways – reaction kinetics (Wittig et al., 2012)), BioCatNet (Buchholz et al., 2016), RetroBioCat‐DB (Finnigan et al., 2023), carbohydrate‐ctive enzymes (Drula et al., 2022)), and commercial ones such as SciFinder or Reaxys. Most current tools in this area remain predictive rather than generative, meaning they assist enzyme discovery and optimization by identifying promising starting points for engineering rather than producing fully novel catalytic sequences de novo.

Selenzyme (Carbonell et al., 2018) began as a cheminformatics‐driven selector that predictively ranks existing candidate enzymes for chemical reactions based on Tanimoto scores between substrate/product and leveraged auxiliary features like phylogenetic distance to the host organism. This fingerprint‐matching strategy made Selenzyme one of the most popular tools in biocatalysis and broadly useful for pathway design. However, the results ultimately depended on how well hand‐crafted fingerprints captured reaction center chemistry, which tends to be incomplete or even ambiguous datasets. The SelenzymeRF (Stoney et al., 2023) upgrade shifts the core similarity metric from fixed reaction fingerprints to ML‐based atom‐to‐atom mapping via RXNMapper. It moves from generic fingerprint overlap to data‐driven identification of the true reaction center, yielding more chemically faithful matches when proposing candidate enzyme sequences.

Enzyme–Substrate Predictor (ESP) is a general ML model able to predict interactions between existing enzyme sequences and substrates, enabling prioritization of likely enzyme–substrate pairs before wet‐lab assays (Kroll et al., 2023). Trained on more than 18,000 experimentally confirmed pairs spanning 1400 metabolites, it reaches >91% accuracy on independent tests where enzymes share ≤80% sequence identity with training data, outperforming family‐specific baselines. The authors note performance drops for metabolites not seen during training, so it is most reliable within the covered metabolite set listed on the server; it is not intended for exhaustive, genome‐scale pair enumeration where false positives would accumulate.

The ESP updated version ProSmith (Kroll et al., 2023) shows stronger generalization to unseen proteins and sparsely represented chemotypes, and typically outperforms ESP on enzyme–substrate benchmarks. As with ESP, its best use is triage: narrowing a focused candidate list before structure‐based modeling and assays; when you have family‐specific measurements, fine‐tuning ProSmith on that set further improves hit rates while still requiring experimental validation for out‐of‐distribution chemistries.

FusionESP (Du et al., 2025), the latest updated and improved version of ESP, is also available for investigating the substrate scope of an enzyme. FusionESP uses a contrastive learning strategy. Compared to ESP, it performs better on enzymes and small molecules which are not part of the training set. Additionally, FusionESP outputs cosine similarity scores to express uncertainty instead of just the positive or negative prediction results.

All‐purpose deep‐learning‐based multiple‐toolkit (ALDELE; (Wang et al., 2024)) is a general deep‐learning toolkit for enzyme screening able to predict whether a given enzyme will catalyze a reaction on a given small molecule, and it also highlights where to engineer by outputting residue‐level hotspot maps and substrate heatmaps. In practice, ALDELE is used to triage enzyme–substrate activity, identify mutational hotspots for engineering, and visualize substrate preferences at low computational cost.

CATNIP (Paton et al., 2025) is a data ML framework that matches substrates to enzymes and vice versa in the α‐ketoglutarate/Fe(II) non‐heme iron family, letting chemists rank likely biocatalysts for a given small molecule or discover plausible substrates for a chosen enzyme. Importantly, the current models and the CATNIP web interface are trained and validated only on this α‐KG/Fe(II) no‐heme iron enzyme family; performance outside this class has not been established and should be considered out of scope unless retrained on appropriate data.

Contrastive learning‐enabled enzyme annotation (CLEAN) is an algorithm for more accurate prediction of the function of uncharacterized enzymes, supporting the discovery of novel enzymes as starting points for engineering (Yu et al., 2023). As an annotation model, CLEAN does not generate sequences directly but can guide downstream design campaigns by identifying functional scaffolds.

Finally, the recently developed EZSpecificity (Zhou & Huang, 2026) predicts enzyme substrate scope by considering 3‐dimensional (3D) structures of binding complexes and using a cross‐attention graph neural network. The enzyme–substrate interaction Gene discovery and enzyme engineering bank dataset built for its training contains enzyme–substrate complex information at sequence, structure, and interaction level, as well as simplified molecular input line entry system (SMILES) of natural and non‐natural substrates. EZSpecificity generates a prediction score for an enzyme's specificity to a substrate molecule. While EZSpecificity performance may be limited if an enzyme–substrate pair lacks active‐site annotations, it can still be applied to find substrates for a target enzyme and rank them by affinity or, the other way around, to find enzymes acting on a specific substrate.

Although predictive models dominate the current landscape, emerging generative protein language models and reaction‐conditioned design frameworks are beginning to address direct sequence creation. These approaches are discussed further in the de novo enzyme design section, where generative modeling is currently more mature.

## 3 AI FOR STRUCTURE PREDICTION

Advances in ML have made protein structure prediction and design much more accurate and accessible in recent years, but these tools have generally been limited to polypeptide chains (Varadi et al., 2025). However, ligands such as small molecules, metal ions, and nucleic acids are crucial components of most proteins, both in terms of structure and biological function. New ML advanced models have now included them, and it is now possible to predict complex enzyme–substrate structures. Here, we provide an overview of leading AI tools for enzyme structure prediction, emphasizing their practical strengths and use cases. A collection of these tools and the repositories where they can be found in Table 2.

**TABLE 2:** Structure prediction artificial intelligence‐tools. A more complete list of prediction methods of protein tertiary conformation has been recently published by Morehead et al. (2026). More details about each model is provided in Table S2.

Model	Description	Repository/webserver	References
AlphaFold3	Predicts the 3D structures and interactions of biomacromolecules	https://alphafoldserver.com/ https://github.com/google‐deepmind/alphafold3	Abramson et al. (2024)
RoseTTAFold All‐Atom	Predicts the 3D structures and interactions of biomacromolecules	https://github.com/baker‐laboratory/RoseTTAFold‐All‐Atom	Krishna et al. (2024)
Chai‐1	Predicts the 3D structures and interactions of biomacromolecules	https://github.com/chaidiscovery/chai‐lab	Chai et al. (2024)
Boltz‐2	Predicts the 3D structures and interactions of biomacromolecules	https://github.com/jwohlwend/boltz	Passaro et al. (2025)

AlphaFold3 (AF3; (Abramson et al., 2024)) is an all‐atom, diffusion‐based structure predictor for general biomolecular complexes, including proteins in complex with DNA/RNA, small‐molecule ligands, ions, or modified (non‐canonical) amino acid residues. Researchers can submit sequence inputs (including multiple protein chains, nucleotide sequences, some ligand specifications, post‐translational modification, and metal ions) to the official server to obtain predicted structures. While AF3 resources are publicly accessible, OpenFold (Ahdritz et al., 2024) remains especially valuable as an open AlphaFold2‐based framework that enables local deployment, retraining, benchmarking, and architectural modification. Therefore, the distinction between both platforms lies less in local availability and more in openness and modifiability.

RoseTTAFold All‐Atom (RFAA) (Krishna et al., 2024) is the Baker Lab's general biomolecular co‐folding model that predicts full assemblies, including proteins complexed with small‐molecule ligands, metals, nucleic acids, and covalent/modified residues. RFAA is fully open‐source and available on GitHub, with full inference code, setup instructions, and a Docker workflow making it generally accessible.

Chai‐1 (Chai et al., 2024) predicts full biomolecular complexes, proteins with small molecules, DNA/RNA, metals, glycans, and modified residues, from simple graphics processing units FASTA plus chemical inputs. Chai‐1 reports 77% success at ligand root mean square deviation (RMSD) ≤2 Å from sequence plus ligand composition/SMILES only, rising when prompted with an apo template. It also shows competitive results on protein–protein and protein‐nucleic‐acid tasks without nucleic‐acid mulitiple sequence aligments. Limitations noted by the authors include occasional mis‐orientation of chains without interfacial restraints and sensitivity to modified residues. Unlike AF3 restricted licensing, Chai‐1 is released as an open model which can be used in commercial research projects, and its code is freely available and includes a webpage friendly application.

Boltz‐2 (Passaro et al., 2025) predicts full biomolecular complexes, proteins with small molecules, DNA/RNA, metals, glycans, and modified or covalent ligands and, crucially, also predicts binding affinity. Boltz‐2 reports a state‐of‐the‐art structure plus affinity accuracy for protein‐ligand systems and strong results across other modalities. Notably, its affinity predictions approach the accuracy of physics‐based free energy perturbation (FEP) while being reported ≈1000× computationally faster, enabling practical in silico screening and iterative design, including substrate affinity. Boltz‐2 is open‐source with code and weights installable via pip for local graphical processing unit runs.

## 4 AI TO SUPPORT ENGINEERING OF SUBSTRATE SPECIFICITY

Each enzyme acts on a specific set of substrate molecules. Changing this substrate scope is one of the main interests in process oriented enzyme engineering as it results in the potential to generate new compounds, which is a significant bottleneck, particularly in pharmaceutical research (Rana et al., 2024). The most basic route to alter the substrate scope is through site‐directed mutagenesis (Wang et al., 2002). However, this approach requires extensive knowledge of protein structure, enzyme–substrate interaction, and structure–function relationship (Wilson & Agard, 1991). Early efforts, for example, required the structure of a homologous enzyme with the desired substrate scope as a template to determine relevant mutations (Wells et al., 1987). Additionally, it is crucial to consider epistasis, a phenomenon describing how the effect of several mutations might not have effects that combine in an additive way (Schultz & Lynch, 1997). The effect might be stronger than just the combination, or the mutations might cancel each other's effects (Starr & Thornton, 2016). Therefore, if done purposefully, large amounts of screening of various mutants are required to change the substrate scope of an enzyme (Zeymer & Hilvert, 2018). A lack of knowledge about protein structure and structure–function relationship, as well as limited screening capacity, remains a challenge in enzyme engineering today; however, this can be tackled with the help of AI tools collected in Table 3 and presented in this section.

**TABLE 3:** Artificial intelligence tools for engineering an enzyme's substrate scope. More details about each model are provided in Table S3.

Model	Description	Experimental validation	Repository/webserver	References
ProGen	Generates novel protein sequences with desired functions	Yes	https://github.com/salesforce/progen	Madani et al. (2023)
PLACER	Predicts binding of enzymes to a substrate	Yes	https://github.com/baker‐laboratory/PLACER	Anishchenko et al. (2025)
EnzyACT	Predicts the impact of mutations on enzyme activity	No	https://github.com/GenScript‐IBDPE/EnzyACT	Li, Zhang, et al. (2024)
AI.zymes	AI‐guided directed evolution of enzymes	Yes	https://github.com/bunzela/AIzymes	Merlicek et al. (2025)
GDEE	Optimizes enzyme sequences to improve desired biological function	No	https://github.com/protein‐modelling‐itqb/gdee	Souza et al. (2025)

**FIGURE 1:**
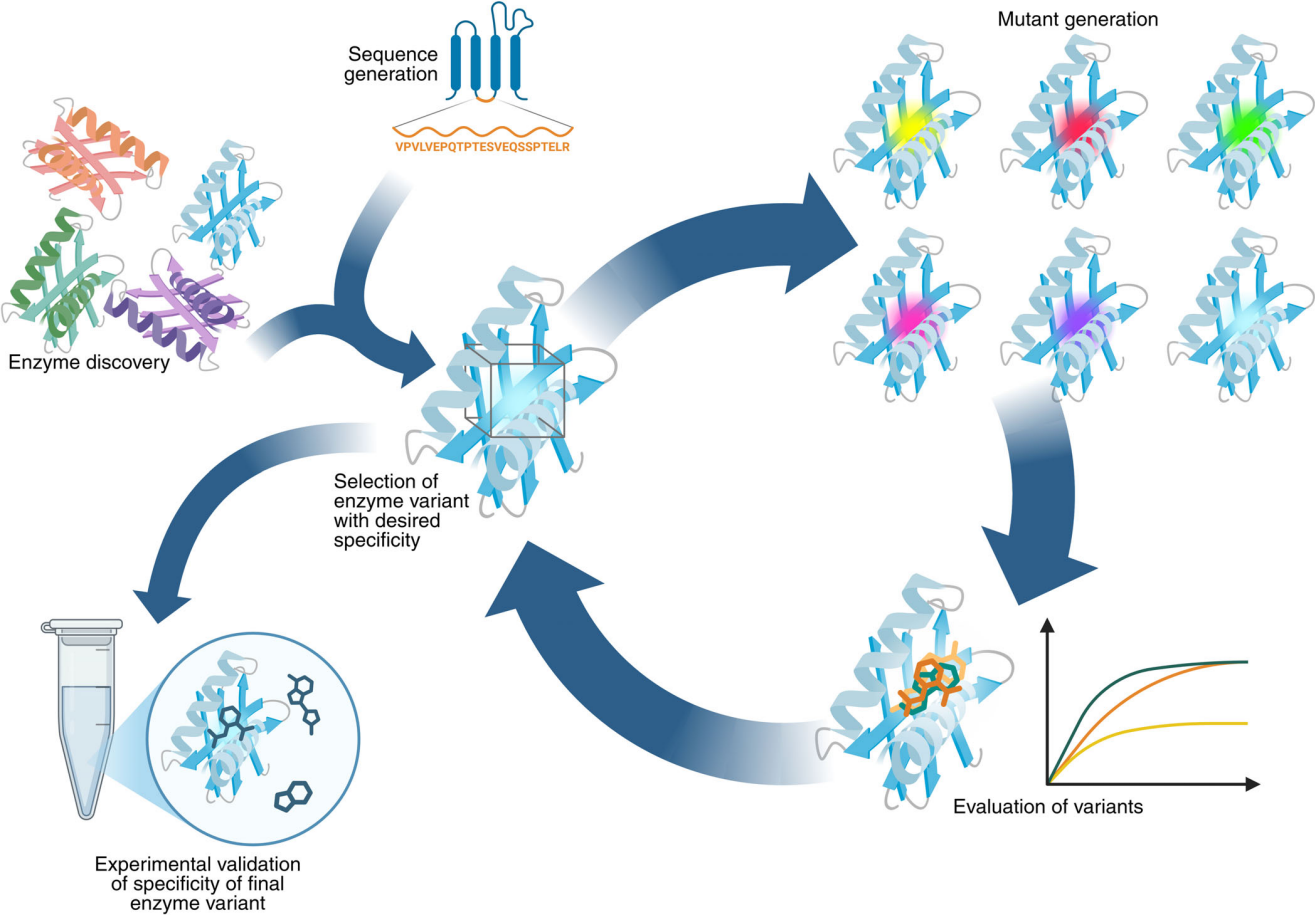
Artificial intelligence‐assisted workflow for enzyme engineering and substrate scope modification. Starting from enzyme discovery or sequence generation, the next step in the pipeline would be to select a suitable variant for the substrate scope studies. Different mutants will be generated in silico and evaluated computationally to optimize the substrate scope. Selected variants will ultimately be subjected to experimental validation. Running multiple rounds of this pipeline, it will be possible to tailor the substrate scope of the original enzyme.

AI tools can support enzyme design at different points of the engineering process (Yang et al., 2024) (Figure 1). For example, ProGen (Madani et al., 2023) is a language model trained with ≈280 million protein sequences from public databases. It can generate new artificial protein sequences with predictable function, homologous to a specific protein family. For example, ProGen was validated on lysozymes after fine‐tuning with a set of lysozyme sequences and testing the expression and functionality of the 100 most promising candidates out of 1 million predictions. Despite low sequence identity, the generated proteins showed similar functionality to natural ones. Therefore, ProGen could be a helpful tool for creating sequences with a desired function as a starting point for engineering efforts toward a specific substrate scope.

After determining the starting enzyme and generating mutants, AI can also support the selection of enzyme variants based on their binding to a desired substrate. Protein‐ligand atomistic conformational ensemble reproduction (PLACER) is a tool that can be applied to assess the accuracy and pre‐organization of a designed active site (Anishchenko et al., 2025).

EnzyACT (Li, Zhang, et al., 2024) is a valuable tool when selecting enzyme variants. EnzyACT can predict the impact of single or multiple mutations on enzyme activity. The deep learning model was trained on a dataset of enzyme mutants, including data about their activity. The activity and stability of an enzyme mutant compared to the wild type can be predicted and considered when choosing an enzyme variant for further engineering.

Besides determining a starting point for engineering and selection variants for the next round of mutagenesis, directed evolution of enzymes can be performed in silico almost entirely with AI.zymes (Merlicek et al., 2025). It combines tools like Rosetta (Kellogg et al., 2011), ESM‐Fold (Lin et al., 2023), ProteinMPNN (Dauparas et al., 2022), and FieldTools in a modular approach to perform the cycles of protein design, selection, and mutation. AI.zymes uses a protein data bank (PDB) coordinate file of the original enzyme as input and outputs a PDB file of the final mutant after iterative rounds of in silico evolutionary design. Therefore, the only remaining experimental work is the validation of the final generated mutants. AI.zymes can be used to engineer substrate scope or other catalytic properties.

Gene discovery and enzyme engineering (GDEE (Souza et al., 2025)) is another recently developed tool for enzyme engineering in silico. The pipeline starts with PDB files of the enzyme and ligand structures. In a first step, mutant sequences are generated by sampling the mutation space for residues the user selects or by generating all possible amino acid combinations for the chosen residues. GDEE can also be used to discover novel enzymes for a specific function; therefore, an input of sequences replaces the mutant generation in the first step, and GDEE will determine the most promising sequence candidates for the desired function.

## 5 AI‐BASED APPROACHES TO AUGMENT PROTEIN STABILITY

**FIGURE 2:**
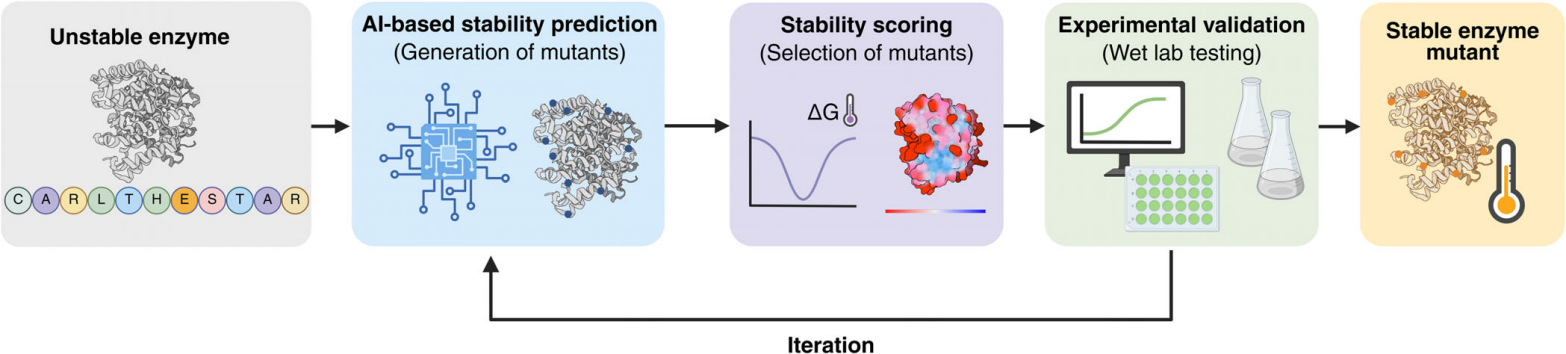
Computational pipeline for the design of stabilized enzymes with artificial intelligence (AI)‐tools. Starting from enzyme variants with low stability, AI‐guided tools such as the ones described in this section can be used to generate stabilized variants.

Achieving simultaneous gains in protein stability and catalytic efficiency remains a central challenge in protein design, as these properties are often intrinsically coupled. While enhanced stability can improve robustness and operational tolerance, it may also restrict the conformational dynamics required for efficient catalysis. The ultimate goal is therefore to design proteins that combine a stable structural framework with the local flexibility and precise active‐site organization necessary for high activity. An ideal biocatalyst should be resistant and robust under diverse experimental conditions (Khan, 2025). However, the applicability of many proteins is affected and thereby limited due to low catalytic rates and/or poor thermostability (Bar‐Even et al., 2011; Goldenzweig et al., 2018). Although the latter bottleneck has been partially addressed with well‐established tools, such as FoldX (Schymkowitz et al., 2005) and Rosetta (Kellogg et al., 2011), it remains challenging to design stable yet active enzyme variants. In recent years, the advancing fields of AI and ML have introduced novel, state‐of‐the‐art models able to predict stabilizing mutations with improved accuracy and performance rates (Figure 2). Here, we describe some of the most recent developments to the best of our knowledge, highlighting the features and output information for each model summarized in Table 4.

**TABLE 4:** Artificial intelligence‐tools to engineer the stability of enzymes. More details about each model are provided in Table S4.

Model	Description	Experimental validation	Repository	References
ProStab	Predicts stabilizing effect of point mutations	Yes	github.com/xtanh/ProStab	Tan et al. (2025)
ProstaNet	Compares structural data to analyze stabilizing effect of mutations	Yes	github.com/NikoBelice/ProstaNet	Liang et al. (2025)
SPURS	Predict how individual mutations affect stability	No	github.com/luo‐group/SPURS	Li and Luo (2025)
Pythia	Assess protein stability based on structure	Yes	github.com/Wublab/pythia	(Sun et al., 2025)
SPIRED‐Stab	Predict protein stability based on sequence	No	github.com/Gonglab‐THU/SPIRED‐Fitness	Chen et al. (2024)
Stability Oracle	Measures chemistry of neighboring atoms and gives information on stability	No	github.com/danny305/StabilityOracle	Diaz et al. (2024)
ESM_therm_	Predicts whether a mutation has stabilizing or destabilizing effects	No	github.com/SimonKitSangChu/EsmTherm	Chu et al. (2024)
ProSTAGE	Predicts structure–function relationship in terms of protein stability	No	github.com/GenScript‐IBDPE/ProSTAGE	Li, Yao, and Fan (2024a)

ProStab (Tan et al., 2025) is one of the latest predicting frameworks reported to date, which provides details on stability changes upon utilization of sequential and structural data of a protein. It yields information about the stabilizing effect of point mutations, avoiding structure predictions of a mutant. The tool has proven its practical usefulness in an experimental study on transaminase, where out of 20 predicted and applied substitutions, four mutants showed increased thermostability.

ProstaNet (Liang et al., 2025) solely relies on structural data of the native protein or its mutants, whose details are then linked together to compare them and extract information on the stabilizing or destabilizing effect of a specific mutation. Its applicability was verified experimentally on humanized nanobody J3 (HuJ3), reaching stability changes of five formerly recognized mutations. Of these, four predictions were consistent with the experimental data.

Another deep learning model which was built on two widely recognized prediction tools is stability prediction using a rewired strategy (SPURS)(Li & Luo, 2025), which incorporates ProteinMPNN (Dauparas et al., 2022) and ESM as structure and sequence‐based frameworks, respectively. Bridging those two models together, SPURS can predict the stability impact of all individual mutations instead of assessing each residue substitution at a time.

Unlike previous supervised learning, Pythia (Sun et al., 2025) stands out as a self‐supervised model that uses protein structures as input data. This protein's stability prediction tool relies exclusively on the protein's structural information, not on the evolutionary context, allowing for the integration of unlabeled data. To assess the experimental validity of the tool, 35 predicted point mutations were tested on limonene epoxide hydrolase, of which 17 resulted in an elevated melting temperature of the enzyme.

Built on a base framework, SPIRED‐Stab (Chen et al., 2024) emerges as an extended model derived from SPIRED (Cui, Su, et al., 2025; Cui, Zheng, et al., 2025a, 2025b) for the prediction of protein stability. In principle, the algorithm predicts the protein structure based on its sequence, which is then fed to SPIRED‐Fitness to evaluate how the one or two substitutions affect the protein fitness in comparison to the native sequence. Consequently, this information is adapted by SPIRED‐Stab to estimate the impact of mutations on protein stability. This tool has not been experimentally validated.

Among these models, Stability Oracle (Diaz et al., 2024) stands out as a framework combining self‐supervised and supervised learning. As a structure‐based predictor, it displays proteins as graphs and measures the chemistry of neighboring atoms at the mutation site, subsequently refining information from this pretrained model on experimentally labeled stability data.

Another model, ESM_therm_ (Chu et al., 2024), was constructed via refinement based on ESM structure predictor, creating a fine‐tuned framework for prediction of protein stability. By extracting information from a curated mega‐scale data collection, it evaluates whether a given mutation poses a stabilizing or destabilizing effect on the protein. ESM_therm_ performs best when predicting mutations on short domains and has strong generalization across distinct proteins.

Lastly, we outline ProSTAGE (Li, Yao, & Fan, 2024), a tool which uses sequential and structural data to predict the effect of point mutations. The proteins are represented in the form of graphs and draw information from interactions between residues, allowing the model to predict structure–function relationships in terms of protein stability.

It is worth noting that the software packages SPURS, SPIRED‐Stab, Stability Oracle, ESM_therm_, and ProSTAGE all lack comparative and quantifiable peer‐reviewed experimental validation, which limits their applicability at this point.

## 6 AI FOR ENZYME DESIGN WITH NON‐CANONICAL AMINO ACIDS

Protein design has been historically limited to the use of the 20 natural amino acids. The incorporation of ncAAs into proteins offers a powerful approach to the expansion of the genetic code, potentially unlocking novel functionalities and improving enzyme performance (Birch‐Price et al., 2024). Despite the increasing interest in designing enzymes containing ncAAs, there is a very limited availability of computational tools adapted to work with them (Hickey et al., 2023; Orr et al., 2022). One of the main problems we face when working on computational design of ncAA‐containing proteins is the limited structural data available, as there are only a few examples of proteins containing ncAAs available in the PDB. Another challenge is the lack of parametrization and structural modeling of side chains differing from the 20 canonical ones. Therefore, in this section we will focus on the description of the available tools and strategies to fill this gap. All tools described here and the repositories where they are stored are collected in Table 5.

**TABLE 5:** Artificial intelligence‐tool for the design of non‐canonical amino acids (ncAA)‐containing proteins. More details about each model are provided in Table S5.

Model	Description	Experimental validation	Repository/webserver	References
NCflow	Predicting biomolecular structure with ncAA	No	Code not available for the moment	Lee and Kim (2025)
RareFold	Structure prediction and design of proteins with ncAAs	Yes	https://github.com/patrickbryant1/RareFold	Li et al. (2025)

It is important to also mention different methods that, while not relying on AI, have helped shape the ncAA‐field, allowing researchers to parametrize different unnatural amino acids and run MD simulations with them. These methods include MakeRotLib (Drew et al., 2013; Renfrew et al., 2012), a library for Rosetta‐basedREF design; FakeRotLib (Bell et al., 2025), a statistical method which allows for the parametrization of more complex ncAAs; ff_NCAA, a force field developed by the Floudas group containing a library of hundreds of parametrized ncAAs compatible with Assisted Model Building with Energy Refinement (Khoury et al., 2014); and the no‐canonical amino acid parameterization toolkit (Case et al., 2025; Overstreet et al., 2024), a semi‐automated approach for ncAA parameter generation for chemistry at HARvard macromolecular mechanics (Brooks et al., 2009) force fields.

**FIGURE 3:**
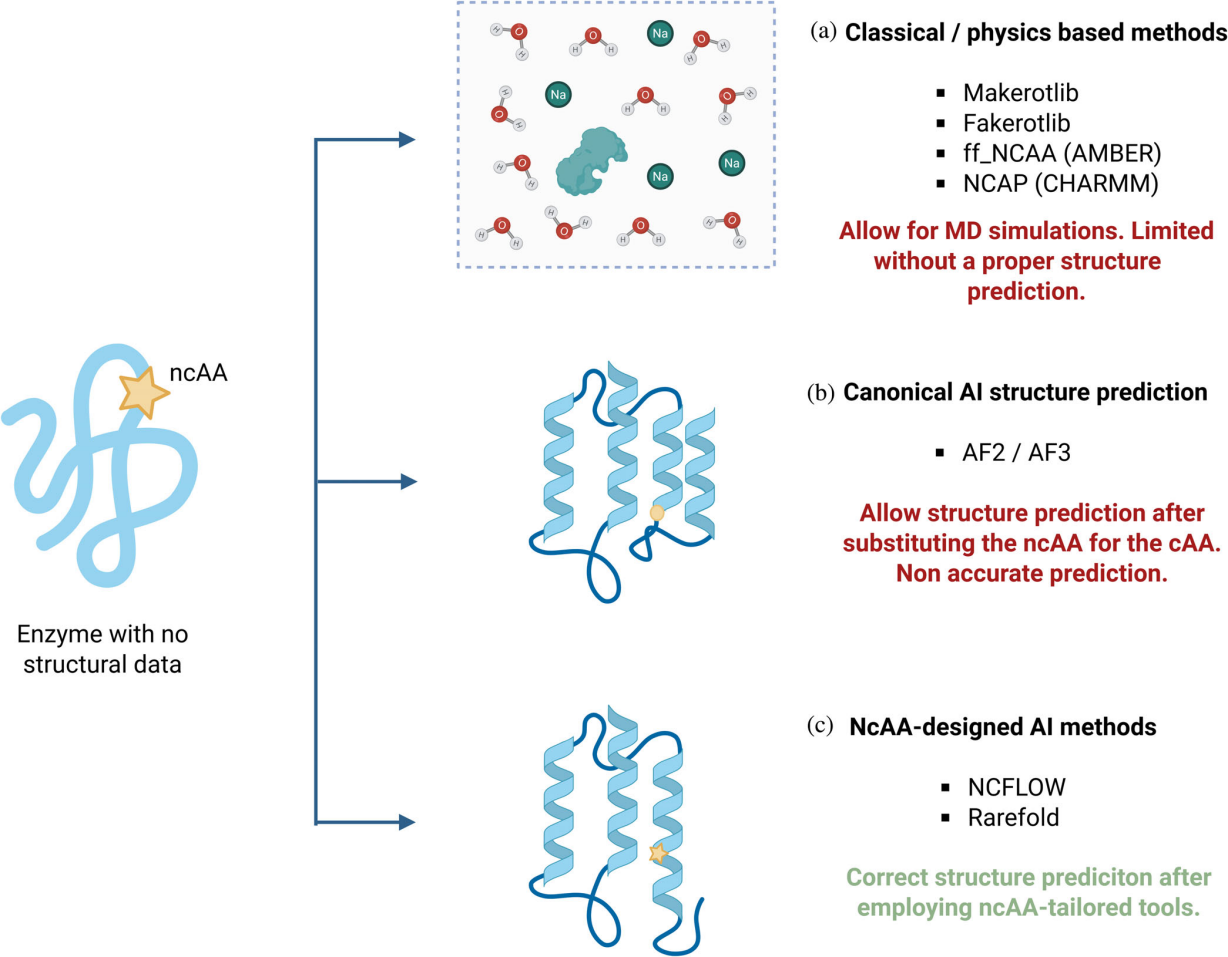
Challenges and new methods for structure prediction of non‐canonical amino acids (ncAA)‐proteins. Classical physics‐based approaches enable the parametrization of ncAA, allowing these unnatural amino acids to be included in molecular dynamics (MD) simulations but are limited to the availability of an initial accurate structure model. Canonical artificial intelligence (AI) structure prediction methods, such as AlphaFold3 (AF3), have not yet implemented parameters to predict structures containing ncAAs. Therefore, the only alternative is to predict the structure of the enzyme containing the closest canonical amino acid (cAA). However, this might lead to inaccurate structural predictions. To overcome this, newly developed AI‐guided methods such as NCFlow or Rarefold have been developed to specifically predict the structure of ncAA‐proteins.

While energy function‐based approaches remain critical, AI‐driven structure prediction tools are increasingly important for modeling proteins, which extend beyond the 20 natural amino acids. Methods such as AF2 and AF3 have revolutionized protein structure prediction, yet they remain inherently limited to the standard set of amino acids encoded in the training data. ncAAs are typically ignored, substituted with the closest canonical residue, or represented with incomplete side‐chain information (Figure 3).

A recent advance is NCFlow (Lee & Kim, 2025), a flow‐matching generative model explicitly developed to handle mixed alphabets of canonical and ncAAs. NCFlow embeds any arbitrary ncAA within a protein chain using an atomic‐graph representation (atoms + bonds rather than fixed residue tokens), thus avoiding reliance on a fixed 20‐amino acid vocabulary. The training follows a three‐stage regimen: first small‐molecule conformations, then protein–ligand complexes, and finally native ncAA residues, enabling the model to learn the rules of chemical space and protein context despite sparse ncAA structure data. In their benchmark tests, the authors show how NCFlow outperforms AF3 in predicting backbone and side‐chain conformations of sequences containing novel ncAAs, achieving lower RMSDs and better side‐chain packing. NCFlow offers a robust and extensible framework, bringing ncAA‐protein design closer to mainstream AI‐guided enzyme engineering, although users must still integrate experimental refinement and validate scaffold‐specific performance.

RareFold (Li et al., 2025) also emerges as a highly promising tool for predicting the structure of ncAA‐containing proteins. Its key innovation lies in its training on the entire Protein Data Bank (PDB) without filtering for sequence similarity, allowing it to learn from a vastly larger and more diverse set of structural motifs. The model is based on the EvoFormer architecture from AF2 but was extended to treat each of the 20 canonical amino acids as well as 29 additional ncAAs as a unique token; it then learns residue‐specific atomic interaction patterns and coevolutionary relationships from a massive, pre‐computed database of protein structures.

More importantly, the RareFold framework can be explicitly fine‐tuned on specific ncAAs. By incorporating a small number of high‐resolution structures containing a target ncAA into its training set, the model can learn the specific geometric and steric properties of that residue. This method was experimentally validated and, while still limited to 29 ncAAs, it is a great advance toward an accurate structure prediction of ncAA‐proteins.

These developments collectively mark important progress toward a more comprehensive computational framework for ncAA‐based enzyme design. Nevertheless, the field remains in an early stage, with challenges including incomplete force‐field coverage, limited training data for AI models, and difficulties integrating ncAAs into end‐to‐end design pipelines.

## 7 AI‐POWERED DE NOVO ENZYME DESIGN PIPELINE

De novo enzyme design conveys an enzyme engineering approach which builds protein scaffolds from scratch around a pre‐defined active site model (Listov & Fleishman, 2026; Tang et al., 2025). This methodology allows researchers to create proteins which do not exist naturally, enabling the design of novel catalytic folds, tailored active sites, and new functions beyond those found in nature. Still, de novo enzyme design remains difficult because designs must consider folding stability, structural integrity, catalytic geometry, dynamics, and substrate binding; in fact, many predicted stable designs still fail experimentally (Kim et al., 2022). Deep learning and generative models are reshaping this field, yet most methods were developed for general protein design rather than enzymes, meaning de novo enzyme design is still in its early stage. Current strategies focus on three main approaches: diffusion‐based backbone generation, hallucination methods, and generative language models; and it is expected that these methods will eventually adapt to enzyme‐specific challenges. Some of the most relevant tools for de novo enzyme design are described in this section and collected in Table 6.

**TABLE 6:** Artificial intelligence‐tools available for de novo design of enzymes.

Model	Description	Experimental validation	Repository/webserver	References
RFDiffusion	De novo diffusion‐based protein design	Yes	https://github.com/RosettaCommons/RFdiffusion	Watson et al. (2023)
RiffDiff	De novo protein design	Yes	https://github.com/mabr3112/riff_diff_protflow	Braun et al. (2026)
ProteinMPNN	Protein sequence design method	Yes	https://github.com/dauparas/ProteinMPNN	Dauparas et al. (2022)
LigandMPNN	Protein sequences optimization to bind specific ligands	Yes	https://github.com/dauparas/LigandMPNN	Dauparas et al. (2025)
GENzyme	Optimizes enzyme sequences for desired biochemical function	No	https://github.com/WillHua127/GENzyme	Hua et al. (2024)
ProtGPT2	De novo sequence‐based protein design	Yes	https://huggingface.co/nferruz/ProtGPT2	Ferruz et al. (2022)

Diffusion models, a class of de‐noising generative models, have become a leading approach for de novo design, as they allow to generate realistic backbone geometries given noisy starting points. Developed by Baker's lab, RFdiffusion (Watson et al., 2023) adapts RoseTTAFold into a backbone de‐noising network, allowing scaffold generation and functional motif grafting under user‐defined structural constraints. Its success has been experimentally validated across various protein types. Since then, diffusion architectures have proliferated, and models like RiffDiff (Braun et al., 2026) are emerging. RiffDiff designs de novo enzymes through a hybrid strategy, which combines ML‐based backbone generation with atomistic modeling to scaffold pre‐defined catalytic arrays. In combination with RFDiffusion and ProteinMPNN it generates a diverse set of stable, de novo enzyme sequences built around a highly potent catalytic core. The strength of these models lies in their ability to sample diverse backbone candidates conditioned on user‐specified structural motifs or design constraints, which is especially useful for catalytic sites requiring precise atomic geometries.

In practice, diffusion models together with sequence design tools such as ProteinMPNN and LigandMPNN (Dauparas et al., 2025) present a commonly used pipeline: first sample candidate backbones with diffusion, then run sequence design (with ProteinMPNN and LigandMPNN), then filter by structural confidence (with AlphaFold (AF) prediction) and energetic validation. This pipeline has already generated experimentally validated de novo designed proteins with completely new functions, such as an artificial metathase designed by Zou et al. (2025). Another case study of a successful de novo generated enzyme is the serine hydrolase designed by Lauko et al. (2025).

In contrast to diffusion methods, which are specifically trained and generate novel sequences by de‐noising, hallucination methods treat protein design as an untrained generative optimization problem (Wicky et al., 2022). Hallucination methods produce novel protein structures without being trained on datasets from that specific target. Instead, they hallucinate designs starting from random noise and iteratively optimizing the target function using neural networks. They define a scoring function and then optimize the inputs to a structure prediction network so that the model hallucinates sequences and structures with high scores (Anishchenko et al., 2021). While there exist multiple methodologies for the use of neural networks, a commonly used approach for de novo protein hallucination feeds the input into trRosetta structure prediction networks (Du et al., 2021; Khakzad et al., 2023; Omar et al., 2023).

Early work demonstrated how networks trained for prediction could generate novel, foldable proteins. Later approaches have combined hallucination with motif scaffolding and function conditioning. The key advantage is flexibility, allowing for the direct optimization of functional metrics that are difficult to encode as structural constraints for diffusion models. However, hallucination is often more sensitive to scoring biases, generating models which adjust well to the scoring function but are actually unrealistic. Hybrid strategies combining diffusion, hallucination, and energy‐based filtering represent an interesting and complete path forward (Du et al., 2021; Hua et al., 2024; Wicky et al., 2022).

Looking ahead, the confluence of these methods is likely to produce integrated design pipelines in which diffusion models generate backbones conditioned on catalytic sites; sequence models would enforce foldability, and hallucination modules would fine‐tune function‐specific metrics like substrate binding affinity or catalytic efficiency. For enzyme design in particular, one promising direction is reaction‐conditioned generative modeling, where the desired chemical transformation acts as a design constraint. One example is the recently proposed model GENzyme (Hua et al., 2024), a reaction‐conditioned, de novo enzyme design framework that takes a catalytic reaction as input (substrate/product SMILES) and generates a catalytic pocket, enzyme (structure plus sequence), and a predicted enzyme–substrate complex. The authors report how this strategy can yield more biologically relevant designs than purely structure or binding‐focused approaches for novel chemistries; code is available and can be run by setting substrate/product SMILES in the configuration and executing the generator to produce candidate enzymes.

Finally, generative language models are data‐driven tools which learn the patterns of natural protein sequences and generate novel enzymes only focusing on sequence, not structure. The Ferruz group developed ProtGPT2 (Ferruz et al., 2022), a transformer‐based model generating novel protein sequences which can then be assessed experimentally. In contrast to purely data‐driven design strategies, the Höcker group has advanced modular and repeat‐based protein design based on rosetta calculations, putting focus on the combination of experimental and computational approaches. A collaboration between both groups experimentally validated their approaches, generating a completely novel protein sequence which was expressed and analyzed, demonstrating that it is foldable and stable (Ferruz et al., 2022).

## 8 CONCLUSIONS

After evaluating the recent advances in computational approaches for enzyme target identification, enzyme scaffolding structural modeling, stability assessment, and enzyme–substrate interactions discussed in this review, it becomes evident that AI is completely reshaping the field of protein design, allowing us to tailor enzymes in ways unimaginable just a few years ago. The integration of ML methods with traditional computational and experimental methods now allows exploration of vast sequence‐structure–function spaces with unprecedented efficiency. Across all major aspects of enzyme engineering, starting with reaction chemistry and structure prediction, to stability optimization, substrate scope analysis, incorporation of ncAAs, and even de novo enzyme generation, AI is redefining the limits of biocatalysis.

Despite these advances, several challenges must still be addressed. Current protein design limits may lie in (1) inability to design highly efficient enzymes de novo, (2) limited understanding of protein dynamics and allostery, (3) inability to accurately model transition states and reaction energetics, (4) difficulty engineering entirely new catalytic mechanisms. The main challenge in enzyme design may not be that we cannot test enough designs; rather, it may be that we do not yet know how to generate sufficiently good designs in the first place. Published studies often report merely successful design candidates, while unsuccessful designs remain largely inaccessible. This publication bias limits the diversity and representativeness of the available training data. Incorporating both positive and negative design outcomes into AI training datasets could substantially enhance model learning by providing information on the factors that govern success and failure, ultimately leading to more robust and predictive design frameworks. Another limitation comes from the current training datasets, which often lack chemical and structural diversity across enzyme families and catalytic mechanisms. Integrating experimental results, both positive and negative, into the design pipeline will be essential to enhance the robustness and interpretability of AI‐generated predictions.

Moreover, the persistent gap between in silico designs and in vitro performance highlights the need for closer integration of computational modeling, MD, and experimental validation pipelines. Hybrid AI frameworks that merge generative models with physics‐based approaches such as MD and quantum mechanics/molecular mechanics (QM/MM) are becoming more relevant and completely changing enzyme engineering approaches for experimentalists. These frameworks can refine designs, matching AI prediction data with accurate energetic evaluations.

In conclusion, the synergy between data‐driven AI methods, MD, and experimental biochemistry will be crucial for the future of enzyme engineering. By focusing on cross‐disciplinary collaboration, the field is moving toward a future where enzyme design will be more accurate and efficient, paving the way for sustainable and innovative biocatalysis.

## AUTHOR CONTRIBUTIONS

**Rosa Teijeiro‐Juiz**, **Nina Egeler**, **Grzegorz Jamróg**, **Marion Ringel**, **Thomas Brück**, **Bruno Di Geronimo**, and **Bernhard Loll** were involved in conceptualization; investigation; visualization; writing—review and editing.

## CONFLICT OF INTEREST STATEMENT

The authors declare that there are no conflicts of interest.

## Supporting information


**Data S1.** Supporting Information.

## Data Availability

All data used in this review are available in publicly accessible repositories.
