# Supplementary material for: Applications and limitations of AI tools in enzyme design
Source: Protein Sci. 2026 Jul 10;35(8):e70698. doi: 10.1002/pro.70698 (PMC13351933; doi:10.1002/pro.70698)
Supplement: Supplementary file 1 — Data S1. Supporting Information. [file PRO-35-e70698-s001.docx]

Supporting information to

**Applications and limitations of AI tools in enzyme design**

Rosa Teijeiro-Juiz^1, ⊥^, Nina Egeler^2, ⊥^, Grzegorz Jamróg^2, ⊥^, Marion Ringel^2^, Thomas Brück^2,^*, Bruno Di Geronimo^3,^*, Bernhard Loll^1,^*

^1^ Institute of Chemistry and Biochemistry, Laboratory of Structural Biochemistry, Freie Universität Berlin; Berlin, 14195, Germany.

^2^ Werner Siemens-Chair of Synthetic Biotechnology, Technical University of Munich (TUM), TUM School of Natural Sciences; Garching, 85748, Germany.

^3^ School of Chemistry and Biochemistry, Georgia Institute of Technology, 901 Atlantic Drive NW, Atlanta, Georgia 30332, United States of America

^⊥^ R.T-J; N.E.; G.J. contributed equally

Corresponding authors

* Thomas Brück - Werner Siemens-Chair of Synthetic Biotechnology, Technical University of Munich (TUM), TUM School of Natural Sciences; Garching, 85748, Germany. Email: brueck@tum.de

* Bruno Di Geronimo - School of Chemistry and Biochemistry, Georgia Institute of Technology, 901 Atlantic Drive NW, Atlanta, Georgia 30332, United States of America. Email: bgeronimo3@gatech.edu

* Bernhard Loll - Institute of Chemistry and Biochemistry, Laboratory of Structural Biochemistry, Freie Universität Berlin; Berlin, 14195, Germany. Email: loll@chemie.fu-berlin.de

**Table S1** AI-driven methods for reaction-to-sequence design.

| **Model** | **Description** | **Method** | **Experimental**  **Validation** | **Repository/Webserver** | **Reference** |
| --- | --- | --- | --- | --- | --- |
| **SelenzymeRF** | Evaluation of enzymes for biochemical reactions and metabolic pathway design | Random forest / reaction mapping | Yes | http://selenzymeRF.synbiochem.co.uk/  https://github.com/synbiochem/selenzyme | (Stoney et al., 2023) |
|  | Input | Target reaction, typically as reaction SMILES / RXN-style reaction representation. Optional metadata can include host organism, pathway context, reaction similarity thresholds, or database filters | | |  |
|  | Output | Ranked CSV/table of enzyme candidates, including enzyme/protein identifiers, source organism, EC annotation, sequence links, and reaction similarity scores | | |  |
| **ESP** | Prediction of  Interactions between enzyme sequences and substrates | Gradient boosting / supervised ML | No | https://esp.cs.hhu.de/  https://github.com/AlexanderKroll/ESP | (Kroll et al., 2023) |
|  | Input | Protein sequence + substrate SMILES | | |  |
|  | Output | A prediction score/probability-like classification output for the enzyme–substrate pair. Related DeepMolecules tools may also predict kinetic parameters | | |  |
| **ProSmith** | Prediction of enzyme-substrate interactions | Multimodal transformer | No | https://github.com/AlexanderKroll/ProSmith | (Kroll et al., 2023) |
|  | Input | CSV files with: Protein sequence, SMILES, and target label | | |  |
|  | Output | Description of the expected interaction between enzyme and substrate prediction with binary/classification score | | |  |
| **FusionESP** | Investigates substrate scope of an enzyme | Contrastive deep learning | No | https://github.com/dzjxzyd/FusionESP  https://rqkjkgpsyu.us-east-1.awsapprunner.com/ | (Du et al., 2025) |
|  | Input | Enzyme sequence + substrate SMILES; The webserver also supports multiple sequences separated by commas and large-scale file upload using .xls, .xlsx, .txt, or FASTA-style inputs | | |  |
|  | Output | Confidence/classification score for whether the enzyme–substrate pair is compatible. The workflow generates protein, molecule, and label embedding files such as df_train_enzy.pt, df_train_smiles.pt, and df_train_label.pt, then reports performance/predictions | | |  |
| **ALDELE** | Predicts if an enzyme will catalyze a reaction | Deep neural network | No | https://github.com/Xiangwen-Wang/ALDELE | (Wang et al., 2024) |
|  | Input | Protein sequences, protein 3D structures, and compound SMILES | | |  |
|  | Output | Predicted enzyme activity value, residue-based hotspots and substrate hotspots | | |  |
| **CATNIP** | Matches substrates to enzymes in α-ketoglutarate/Fe(II) non-heme iron family | Family-specific supervised ML | No | https://catnip.cheme.cmu.edu/ | (Paton et al., 2025) |
|  | Input | Either substrate input to rank compatible enzymes, or enzyme sequence input to rank compatible substrates | | |  |
|  | Output | Ranked predictions of compatible enzyme/substrate pairs | | |  |
| **CLEAN** | Enzyme function prediction | Contrastive learning model | Yes | https://github.com/tttianhao/CLEAN  https://clean.frontend.mmli1.ncsa.illinois.edu/configuration | (Yu et al., 2023) |
|  | Input | Enzyme fasta sequence | | |  |
|  | Output | CSV output with EC number of the target enzyme | | |  |
| **EZSpecifity** | Predicts substrates an enzyme is likely to act on | Cross-attention graph neural network | No | https://zenodo.org/records/17981381 | (Cui et al., 2025) |
|  | Input | Enzyme fasta sequence + substrate SMILES | | |  |
|  | Output | EZSpecificity score or ranked compatibility prediction for enzyme–substrate specificity | | |  |

**Table S2** Structure prediction AI-tools.

| **Model** | **Description** | **Method** | **Repository/Webserver** | **Reference** |
| --- | --- | --- | --- | --- |
| **AlphaFold3** | Predicts the 3D structures and interactions of biomacromolecules | Diffusion deep learning | https://alphafoldserver.com/  https://github.com/google-deepmind/alphafold3 | (Abramson et al., 2024) |
|  | Input | Protein sequence, DNA/RNA, ligands (restricted) and ions | |  |
|  | Output | Predicted mmCIF models, plus confidence metrics pLDDT, PAE, pTM, ipTM | |  |
| **RoseTTAFold All-Atom** | Predicts the 3D structures and interactions of biomacromolecules | Neural network / Deep learning | https://github.com/baker-laboratory/RoseTTAFold-All-Atom | (Krishna et al., 2024) |
|  | Input | Protein sequence, DNA/RNA, covalent modifications and ligands | |  |
|  | Output | Predicted PDB/mmCIF-style structures and model-quality/error estimates | |  |
| **Chai-1** | Predicts the 3D structures and interactions of biomacromolecules | Diffusion multimodal generative model | https://github.com/chaidiscovery/chai-lab | (Chai et al., 2024) |
|  | Input | Protein sequence + ligands | |  |
|  | Output | Predicted 3D structures CIF output and confidence-related outputs such as pLDDT-like | |  |
| **Boltz-2** | Predicts the 3D structures and interactions of biomacromolecules | Diffusion-based generative model | https://github.com/jwohlwend/boltz | (Passaro et al., 2025) |
|  | Input | Protein fasta sequence, DNA/RNA and ligands; MSA/template/configuration settings can be added | |  |
|  | Output | Predicted 3D structure and confidence outputs and affinity values (FEP) between protein and ligand | |  |

**Table S3** AI tools for engineering an enzyme's substrate scope.

| **Model** | **Description** | **Method** | **Experimental Validation** | **Repository/Webserver** | **Reference** |
| --- | --- | --- | --- | --- | --- |
| **ProGen** | Generates novel protein sequences with desired functions | Large language model | Yes | https://github.com/salesforce/progen | (Madani et al., 2023) |
|  | Input | Control/context tokens or tags, such as taxonomic labels, functional keywords, molecular-function annotations, or partial sequence context | | |  |
|  | Output | Protein fasta sequences of enzyme predicted to fit in the control tags categories | | |  |
| **PLACER** | Predicts binding of enzymes to a substrate | Deep learning | Yes | https://github.com/baker-laboratory/PLACER | (Anishchenko et al., 2025) |
|  | Input | Input PDB/mmCIF containing the protein and ligand already present, plus optional ligand connectivity information via SDF/MOL2/CCD, number of samples, crop/corruption centers, fixed ligand, predicted ligand, and ranking metric | | |  |
|  | Output | Multiple PDB structural models of the local protein–ligand complex, plus CSV score file | | |  |
| **EnzyACT** | Predicts the impact of mutations on enzyme activity | ML | No | https://github.com/GenScript-IBDPE/EnzyACT | (Li et al., 2024) |
|  | Input | Protein fasta sequence | | |  |
|  | Output | Predicted activity-change score/classification for each mutant, whether the mutation is likely to increase, decrease relative activity compare it with the wild type | | |  |
| **AI.zymes** | AI-guided directed evolution of enzymes | Generative algorithms | Yes | https://github.com/bunzela/AIzymes | (Merlicek et al., 2025) |
|  | Input | PDB file defining the target residues/active-site region, and optional substrate | | |  |
|  | Output | Designed enzyme variants **in** PDB format with with ranking metrics | | |  |
| **GDEE** | Optimizes enzyme sequences to improve desired biological function | Generative deep learning | No | https://github.com/protein-modelling-itqb/gdee | (Souza et al., 2025) |
|  | Input | Enzyme 3D structure PDB, sequence/FASTA or BLAST-derived homologs, target mutation positions and ligand structure (PDBQT), docking parameters and target mutations | | |  |
|  | Output | SQLite database with ranked mutants based on docking scores | | |  |

**Table S4** AI-tools to engineer stability of enzymes.

| **Model** | **Description** | **Method** | **Experimental**  **validation** | **Repository** | **Reference** |
| --- | --- | --- | --- | --- | --- |
| **ProStab** | Predicts stabilizing effect of point mutations | ML | Yes | github.com/xtanh/ProStab | (Tan et al., 2025) |
|  | Input | Wild-type and mutant protein sequence + wild-type structure | | |  |
|  | Output | ∆∆G prediction based on mutations | | |  |
| **ProstaNet** | Compares structural data to analyze stabilizing effect of mutations | Deep learning | Yes | github.com/NikoBelice/ProstaNet | (Liang et al., 2025) |
|  | Input | Wild-type fasta sequence, mutant fasta sequences and wild-type structure | | |  |
|  | Output | ΔΔG prediction for each mutation | | |  |
| **SPURS** | Predict how individual mutations affect stability | ML | No | github.com/luo-group/SPURS | (Li & Luo, 2025) |
|  | Input | Protein fasta sequence | | |  |
|  | Output | ∆∆G prediction based on mutations | | |  |
| **Pythia** | Assess protein stability based on structure | Self- supervised | Yes | github.com/Wublab/pythia | (Sun et al., 2025) |
|  | Input | Protein 3D structure in PDB format | | |  |
|  | Output | ∆∆G prediction based on mutations | | |  |
| **SPIRED-Stab** | Predict protein stability based on sequence | ML | No | github.com/Gonglab-THU/SPIRED-Fitness | (Chen et al., 2024) |
|  | Input | Protein sequence | | |  |
|  | Output | Predicted protein stability metrics, ΔΔG and ΔTm for mutations | | |  |
| **Stability Oracle** | Measures chemistry of neighboring atoms and gives information on stability | ML | No | github.com/danny305/StabilityOracle | (Diaz et al., 2024) |
|  | Input | Protein 3D structure (PDB) | | |  |
|  | Output | Prediction of changes in ∆∆G for point mutations as well as deep mutational scanning | | |  |
| **ESM_therm_** | Predicts whether a mutation has stabilizing or destabilizing effects | Large language model | No | github.com/SimonKitSangChu/EsmTherm | (Chu, Narang, Siegel, 2024) |
|  | Input | Protein sequence | | |  |
|  | Output | Predicted folding stability score / ΔG-like stability value | | |  |
| **ProSTAGE** | Predicts structure-function relationship in terms of protein stability | ML | No | github.com/GenScript-IBDPE/ProSTAGE | (Li, Yao, Fan, 2024) |
|  | Input | Wild-type and mutant protein fasta sequences | | |  |
|  | Output | Predicted ΔΔG value for the mutation | | |  |

**Table S5** AI-tool for the design of ncAA-containing proteins.

| **Model** | **Description** | **Method** | **Experimental**  **Validation** | **Repository/Webserver** | **Reference** |
| --- | --- | --- | --- | --- | --- |
| **NCflow** | Predicting biomolecular structure with ncAA | Flow-matching generative model | No | Code not available for the moment | (Lee & Kim, 2025, Li et al., 2025) |
|  | Input | Protein-peptide complex structural information and ncAA SMILES | | |  |
|  | Output | Predicted 3D coordinates of the ncAA-containing residue in the local pocket or peptide environment; potentially a modified protein structure containing the requested ncAA | | |  |
| **RareFold** | Structure prediction and design of proteins with ncAAs | Deep learning | Yes | https://github.com/patrickbryant1/RareFold | (Li et al., 2025) |
|  | Input | Protein fasta sequence and ncAA token | | |  |
|  | Output | Predicted 3D structure of the designed cyclic peptide/binder with ncAAs | | |  |

**Table S6** AI-tools available for de novo design of enzymes.

| **Model** | **Description** | **Experimental Validation** | **Repository/Webserver** | **Reference** |
| --- | --- | --- | --- | --- |
| **RFDiffusion** | *De novo* diffusion-based protein design | Yes | https://github.com/RosettaCommons/RFdiffusion | (Watson et al., 2023) |
|  | Input | Contig/design constraints, such as target length, chain breaks, symmetry/copies, fixed motif residues, target protein structure for binder design, optional scaffold constraints, optional catalytic/functional motif information or full protein 3D structure | |  |
|  | Output | PDB files containing generated backbone structures (poly Gly), often followed by ProteinMPNN/LigandMPNN sequence design | |  |
| **RiffDiff** | *De novo* protein design | Yes | https://github.com/mabr3112/riff_diff_protflow | (Braun et al., 2026) |
|  | Input | \| Theozyme/catalytic motif PDB, catalytic residue list, ligand residue information, and design settings \| \| --- \| | |  |
|  | Output | PDB files of designed enzyme scaffolds/variants, usually containing the placed catalytic motif and ligand context, followed by downstream sequence design/filtering in ProtFlow-style workflows | |  |
| **ProteinMPNN** | Protein sequence design method | Yes | https://github.com/dauparas/ProteinMPNN | (Dauparas et al., 2022) |
|  | Input | PDB file protein backbone, with optional fixed positions/chains and temperature (different models might loaded, e.g. solubleMPNN) | |  |
|  | Output | Amino acid sequence (FASTA file) which will fit the provided 3D structure, recovering sequence percentage and proteinMPNN scores | |  |
| **LigandMPNN** | Protein sequences optimization to bind specific ligands | Yes | https://github.com/dauparas/LigandMPNN | (Dauparas et al., 2025) |
|  | Input | PDB structure containing the protein backbone and ligand/non-protein atoms, with optional fixed residues, ligand masks, chain design settings, side-chain packing options and temperature (different models might loaded, e.g. solubleMPNN) | |  |
|  | Output | Amino acid sequence (FASTA file) that will fold into the provided scaffold and bind provided ligand, sequence recovering with overall and ligand confidence | |  |
| **GENzyme** | Optimizes enzyme sequences for desired biochemical function | No | https://github.com/WillHua127/GENzyme | (Hua et al., 2024) |
|  | Input | Target catalytic reaction using substrate and product SMILES format | |  |
|  | Output | Structural prediction of the enzyme-substrate complex and novel protein/pocket sequence | |  |
| **ProtGPT2** | *De novo* sequence-based protein design | Yes | https://huggingface.co/nferruz/ProtGPT2 | (Ferruz, Schmidt, Höcker, 2022) |
|  | Input | Protein sequence / Sequence fragments | |  |
|  | Output | Full *de novo* protein fasta sequence | |  |

**References**

Abramson, J., Adler, J., Dunger, J., Evans, R., Green, T., Pritzel, A., Ronneberger, O., Willmore, L., Ballard, A. J., Bambrick, J., Bodenstein, S. W., Evans, D. A., Hung, C. C., O'Neill, M., Reiman, D., Tunyasuvunakool, K., Wu, Z., Zemgulyte, A., Arvaniti, E., Beattie, C., Bertolli, O., Bridgland, A., Cherepanov, A., Congreve, M., Cowen-Rivers, A. I., Cowie, A., Figurnov, M., Fuchs, F. B., Gladman, H., Jain, R., Khan, Y. A., Low, C. M. R., Perlin, K., Potapenko, A., Savy, P., Singh, S., Stecula, A., Thillaisundaram, A., Tong, C., Yakneen, S., Zhong, E. D., Zielinski, M., Zidek, A., Bapst, V., Kohli, P., Jaderberg, M., Hassabis, D., Jumper, J. M. "Addendum: Accurate structure prediction of biomolecular interactions with AlphaFold 3." Nature 2024; 636(8042):E4.

Anishchenko, I., Kipnis, Y., Kalvet, I., Zhou, G., Krishna, R., Pellock, S. J., Lauko, A., Lee, G. R., An, L., Dauparas, J., DiMaio, F., Baker, D. "Modeling protein-small molecule conformational ensembles with PLACER." Proc Natl Acad Sci U S A 2025; 122(45):e2427161122.

Braun, M., Tripp, A., Chakatok, M., Kaltenbrunner, S., Fischer, C., Stoll, D., Bijelic, A., Elaily, W., Totaro, M. G., Moser, M., Hoch, S. Y., Lechner, H., Rossi, F., Aleotti, M., Hall, M., Oberdorfer, G. "Computational enzyme design by catalytic motif scaffolding." Nature 2026; 649(8095):237-245.

Chai, D., Boitreaud, J., Dent, J., McPartlon, M., Meier, J., Reis, V., Rogozhnikov, A., Wu, K. "Chai-1: Decoding the molecular interactions of life." bioRxiv 2024:2024.2010.2010.615955.

Chen, Y. H., Xu, Y. X., Liu, D., Xing, Y. G., Gong, H. P. "An end-to-end framework for the prediction of protein structure and fitness from single sequence." Nature Communications 2024; 15(1)

Chu, S. K. S., Narang, K., Siegel, J. B. "Protein stability prediction by fine-tuning a protein language model on a mega-scale dataset." Plos Computational Biology 2024; 20(7)

Cui, H., Su, Y., Dean, T. J., Yu, T., Zhang, Z., Peng, J., Shukla, D., Zhao, H. "Enzyme specificity prediction using cross-attention graph neural networks." Nature 2025; 647(8090):639-647.

Dauparas, J., Anishchenko, I., Bennett, N., Bai, H., Ragotte, R. J., Milles, L. F., Wicky, B. I. M., Courbet, A., de Haas, R. J., Bethel, N., Leung, P. J. Y., Huddy, T. F., Pellock, S., Tischer, D., Chan, F., Koepnick, B., Nguyen, H., Kang, A., Sankaran, B., Bera, A. K., King, N. P., Baker, D. "Robust deep learning-based protein sequence design using ProteinMPNN." Science 2022; 378(6615):49-56.

Dauparas, J., Lee, G. R., Pecoraro, R., An, L., Anishchenko, I., Glasscock, C., Baker, D. "Atomic context-conditioned protein sequence design using LigandMPNN." Nat Methods 2025; 22(4):717-723.

Diaz, D. J., Gong, C., Ouyang-Zhang, J., Loy, J. M., Wells, J., Yang, D., Ellington, A. D., Dimakis, A. G., Klivans, A. R. "Stability Oracle: a structure-based graph-transformer framework for identifying stabilizing mutations." Nat Commun 2024; 15(1):6170.

Du, Z. J., Fu, W. M., Guo, X. L., Caragea, D., Li, Y. H. "FusionESP: Improved Enzyme-Substrate Pair Prediction by Fusing Protein and Chemical Knowledge." Journal of Chemical Information and Modeling 2025; 65(6):2806-2817.

Ferruz, N., Schmidt, S., Höcker, B. "ProtGPT2 is a deep unsupervised language model for protein design." Nat Commun 2022; 13(1):4348.

Hua, C., Lu, J., Liu, Y., Zhang, O., Tang, J., Ying, R., Jin, W., Wolf, G., Precup, D., Zheng, S. (2024). Reaction-conditioned *de novo* Enzyme Design with GENzyme.

Krishna, R., Wang, J., Ahern, W., Sturmfels, P., Venkatesh, P., Kalvet, I., Lee, G. R., Morey-Burrows, F. S., Anishchenko, I., Humphreys, I. R., Mchugh, R., Vafeados, D., Li, X. T., Sutherland, G. A., Hitchcock, A., Hunter, C. N., Kang, A. L., Brackenbrough, E., Bera, A. K., Baek, M., Dimaio, F., Baker, D. "Generalized biomolecular modeling and design with RoseTTAFold All-Atom." Science 2024; 384(6693)

Kroll, A., Ranjan, S., Engqvist, M. K. M., Lercher, M. J. "A general model to predict small molecule substrates of enzymes based on machine and deep learning." Nat Commun 2023; 14(1):2787.

Lee, J. S., Kim, P. M. "Design of peptides with non-canonical amino acids using flow matching." bioRxiv 2025;

Li, G., Yao, S. J., Fan, L. "ProSTAGE: Predicting Effects of Mutations on Protein Stability by Using Protein Embeddings and Graph Convolutional Networks." Journal of Chemical Information and Modeling 2024; 64(2):340-347.

Li, G., Zhang, N., Dai, X. W., Fan, L. "EnzyACT: A Novel Deep Learning Method to Predict the Impacts of Single and Multiple Mutations on Enzyme Activity." Journal of Chemical Information and Modeling 2024; 64(15):5912-5921.

Li, Q., Daumiller, D., Zuo, F., Marcotte, H., Pan-Hammarström, Q., Bryant, P. "RareFold: Structure prediction and design of proteins with noncanonical amino acids." bioRxiv 2025;

Li, Z., Luo, Y. "Rewiring protein sequence and structure generative models to enhance protein stability prediction." bioRxiv 2025:2025.2002.2013.638154.

Liang, T. J., Sun, Z. Y., Ishima, R., Xie, X. Q., Xue, Y., Li, W., Feng, Z. W. "ProstaNet: A Novel Geometric Vector Perceptrons-Graph Neural Network Algorithm for Protein Stability Prediction in Single- and Multiple-Point Mutations with Experimental Validation." Research 2025; 8

Madani, A., Krause, B., Greene, E. R., Subramanian, S., Mohr, B. P., Holton, J. M., Olmos, J. L., Jr., Xiong, C., Sun, Z. Z., Socher, R., Fraser, J. S., Naik, N. "Large language models generate functional protein sequences across diverse families." Nat Biotechnol 2023; 41(8):1099-1106.

Merlicek, L. P., Neumann, J., Lear, A., Degiorgi, V., de Waal, M. M., Cotet, T. S., Mulholland, A. J., Bunzel, H. A. "AI.zymes: A Modular Platform for Evolutionary Enzyme Design." Angew Chem Int Ed Engl 2025; 64(27):e202507031.

Passaro, S., Corso, G., Wohlwend, J., Reveiz, M., Thaler, S., Somnath, V. R., Getz, N., Portnoi, T., Roy, J., Stark, H., Kwabi-Addo, D., Beaini, D., Jaakkola, T., Barzilay, R. "Boltz-2: Towards Accurate and Efficient Binding Affinity Prediction." bioRxiv 2025:2025.2006.2014.659707.

Paton, A. E., Boiko, D. A., Perkins, J. C., Cemalovic, N. I., Reschützegger, T., Gomes, G., Narayan, A. R. H. "Connecting chemical and protein sequence space to predict biocatalytic reactions." Nature 2025; 646(8083)

Souza, C. S., Correia, J. P. G., Rocha, I., Lousa, D., Soares, C. M. "GDEE: A Structure-Based Platform for Gene Discovery and Enzyme Engineering." bioRxiv 2025;

Stoney, R. A., Hanko, E. K. R., Carbonell, P., Breitling, R. "SelenzymeRF: updated enzyme suggestion software for unbalanced biochemical reactions." Computational and Structural Biotechnology Journal 2023; 21:5868-5876.

Sun, J., Zhu, T., Cui, Y., Wu, B. "Structure-based self-supervised learning enables ultrafast protein stability prediction upon mutation." Innovation (Camb) 2025; 6(1):100750.

Tan, H., Wei, X., Lin, S., Mao, X., Chen, J., Sun, H., Zhang, Y., Zhou, Z., Wei, D.-Q., Lin, S., Xiong, Y. "ProStab: Prediction of protein stability change upon mutations by protein language and inverse folding models." bioRxiv 2025:2025.2008.2011.669595.

Wang, X. W., Quinn, D., Moody, T. S., Huang, M. L. "ALDELE: All-Purpose Deep Learning Toolkits for Predicting the Biocatalytic Activities of Enzymes." Journal of Chemical Information and Modeling 2024; 64(8):3123-3139.

Watson, J. L., Juergens, D., Bennett, N. R., Trippe, B. L., Yim, J., Eisenach, H. E., Ahern, W., Borst, A. J., Ragotte, R. J., Milles, L. F., Wicky, B. I. M., Hanikel, N., Pellock, S. J., Courbet, A., Sheffler, W., Wang, J., Venkatesh, P., Sappington, I., Torres, S. V., Lauko, A., De Bortoli, V., Mathieu, E., Ovchinnikov, S., Barzilay, R., Jaakkola, T. S., DiMaio, F., Baek, M., Baker, D. "De novo design of protein structure and function with RFdiffusion." Nature 2023; 620(7976):1089-1100.

Yu, T., Cui, H., Li, J. C., Luo, Y., Jiang, G., Zhao, H. "Enzyme function prediction using contrastive learning." Science 2023; 379(6639):1358-1363.
